# Identifying predictive factors for long-term visual recovery after corneal endothelial keratoplasty in Fuchs' dystrophy: Potential interaction between the corneal dysfunction and retinal status

**DOI:** 10.3389/fmed.2023.1120283

**Published:** 2023-03-09

**Authors:** Charlotte Maffre, Pierre Fournié, Eve Durbant, Carl Arndt, Zoubir Djerada, Alexandre Denoyer

**Affiliations:** ^1^University of Reims Champagne-Ardenne, Reims, France; ^2^University Hospital Robert Debré, Reims, France; ^3^University of Toulouse III Paul Sabatier, Toulouse, France; ^4^CARDIOVIR Research Team, EA-4684, University of Reims Champagne-Ardenne, Reims, France

**Keywords:** cornea, Descemet membrane endothelial keratoplasty, Fuchs, cornea guttata, predictive model

## Abstract

**Introduction:**

Descemet membrane endothelial keratoplasty (DMEK) is the main treatment for Fuchs' dystrophy (FECD). The outcomes are excellent, but the final visual recovery may vary from patient to patient with sometimes no obvious reason of such a spread.

**Methods:**

We conducted a clinical prospective multicentric study to identify the predictive factors for the visual result 1 year after surgery. Eighty three patients (83 eyes) were included.

**Results:**

Postoperative BCVA after 1 year was 0.20 ± 0.18 logMAR. Logistic regression revealed that good visual recovery correlated negatively with preoperative central macular thickness (*p* < 0.001) and the need for rebubbling (*p* = 0.05), and positively with preoperative visual acuity (*p* = 0.009). Multivariate formula to predict the 1-year BCVA has been suggested.

**Discussion:**

Preoperative retinal status seems to be the main predictive factor for long-term visual result after DMEK. Our predictive multivariate model could assist in better informing the patient about the prognosis of the surgery, and in adjusting the therapeutic strategy also, further highlighting the essential collaboration between both cornea and retina subspecialists.

## Introduction

Worldwide, Fuchs' endothelial corneal dystrophy (FECD) is the most common corneal dystrophy (1). It is a bilateral and partially hereditary chronic disease characterized by progressive dysfunction and loss of corneal endothelial cells. FECD may lead to corneal edema, resulting in blurred vision, loss of best corrected visual acuity (BCVA), and, sometimes, painful epithelial damage (2). Specific surgical procedures have been developed over the past decade based on the targeted replacement of damaged tissue. As a result, penetrating keratoplasty was replaced by Descemet stripping automated endothelial keratoplasty (DSAEK) and, more recently, by Descemet membrane endothelial keratoplasty (DMEK), which targets the patient's membrane and endothelial cell layer (3). In addition to the low rate of surgical complications and graft rejection of DMEK when compared with other techniques, most previous studies also attributed excellent outcomes to DMEK (4, 5).

In our experience, however, visual recovery after DMEK may sometimes vary from patient to patient, even in cases that are apparently similar. This may lead to a lack of predictability in the final visual outcomes after this procedure. Previous studies have considered the factors influencing the outcomes of DMEK. Brockmann et al. (6) investigated BCVA, central corneal thickness, and endothelial cell density after DMEK in 108 eyes with Fuchs' dystrophy. They reported that a corneal thickness, as measured by pachymetry, >625 μm is significantly linked to poor BCVA after the surgery. As a result, a cut-off was defined when giving advice relating to performing DMEK. Schrittenlocher et al. (7) found that a preoperative BCVA below 20/100 indicated poor visual recovery after 12 months, probably due to structural alterations in the cornea occurring in the late stages of the disease. Other factors, including corneal haze and graft–host irregularity, were highlighted by Turnbull et al. (8).

Considering the retina, Zwingelber et al. reported that vitreomacular traction may influence the visual prognosis (9), and Steindor et al. studied DMEK outcomes in patients with macular comorbidities (10). However, poor things are known about relationship between the corneal and the putative retinal changes in this disease. The purpose of this prospective cohort study was to provide a global multivariate model to better understand and predict the long-term visual recovery after DMEK in the treatment of FECD.

## Materials and methods

### Study design

This prospective observational multicentric study was conducted in the Quinze-Vingts National Ophthalmology Hospital (Paris, France; CIC 503, CPP Île-de-France, agreement number 10793), the department of ophthalmology at the Purpan University Hospital (Toulouse, France), and the department of ophthalmology at the Robert Debré University Hospital (Reims, France), in accordance with the Declaration of Helsinki, Scotland Amendment, 2000. Permission was obtained from the institutional review board, and all patients gave validated and informed consent.

From January 2018 to September 2019, patients scheduled for DMEK were consecutively enrolled. Fuchs' dystrophy requiring DMEK were included according to the main criteria for surgery, i.e., fluctuating vision throughout the day, photophobia, BCVA > 0.4 logarithm of the minimum angle of resolution (logMAR), and clinical corneal edema. The main exclusion criteria were any ocular disease (ocular surface disease, inflammatory or infectious diseases, intraocular pressure >21 mmHg or diagnosed glaucoma, retinal disease) aside from Fuchs' dystrophy and cataract. Patients with macular edema (CMT >300 μm) without any other retinal abnormality were not excluded. Other exclusion criteria were age < 21 years, pregnancy, or an inability to understand the study and give informed consent.

Patients' preoperative data were collected: age; gender; symptoms including photophobia or morning fog; diabetic status; distant BCVA (logMAR scale); keratometry; refractive spherical equivalent, intraocular pressure (IOP); optical coherence tomography (OCT) corneal thickness mapping (RT-Vue™, Optovue, Fremont, USA); OCT foveal thickness; and macular status (Spectralis-OCT™, Sanotek, Heidelberg, Germany). Graft features were also recorded, including endothelial cell density, donor's age and gender, as well as the need for rebubbling.

All patients underwent DMEK under general anesthesia, which was performed by three experienced surgeons, following a standard “no touch” method, as described by G Melles (11, 12). No iridotomy was performed during the surgery. Postoperatively, patients were placed in a supine position for 48 h, and pharmacological mydriasis has been maintained for the day of the surgery using phenylephrine and tropicamide. Rebubbling was decided in cases with persistent edema and a partially detached graft, as imaged by OCT. Rebubbling was performed within the 7 days after DMEK. All patients received treatment with dexamethasone eye drops postoperatively (tid for 6 months, bid for 3 months, and then once a day) and tear-film substitutes.

The following postoperative data were collected 1 year after the surgery: BCVA, keratometry, OCT corneal thickness mapping, and OCT macular thickness mapping. One-year distant BCVA was the primary endpoint.

### Statistical analysis

All data are given as mean ± SD. Data were controlled for normality and homogeneity of variance to perform adequate tests. The probability level of significance was adjusted according to the *post-hoc* Bonferroni procedure to maintain an overall type I error of 0.05.

For binomial analysis, the patients were separated into two groups depending on their distant BCVA (long-term visual recovery). To facilitate analysis, we choose a score of 1 or 0 for binomial factors. Visual recovery was considered to be good if it was < 0.4 logMAR or bad if it was ≥0.4 logMAR, accordingly to the inclusion criteria and the results previously published about post-DMEK visual recovery (13). *T*-tests, chi-square tests, and scatter plots with the R^2^ coefficient were performed to assess the univariate associations between pairs of variables. For multivariate analysis, the a priori power was estimated using the number of events. This allowed us to include up to nine covariates in the multivariate analysis, with a power >80%. The *post-hoc* simulations showed that the power of all multivariate analyses was higher than 90%. Variables included in the full model were selected using a step-down model. All the selected variables were checked to ensure that no collinearity existed between them. We used the bootstrap method (*n* = 500) to study the uncertainty in the selected variables and to penalize this uncertainty when estimating the predictive performance of the model. The R^2^ indexes were used as discrimination indicators, and the C statistics and Dxy indexes were used as rank discrimination indicators.

The statistical analysis resulted in two attractive models, one with logistic regression and one with multiple linear regression, allowing us to suggest computation formulae to predict the distant BCVA 1 year postoperatively.

## Results

### Population

**Figure 1:**
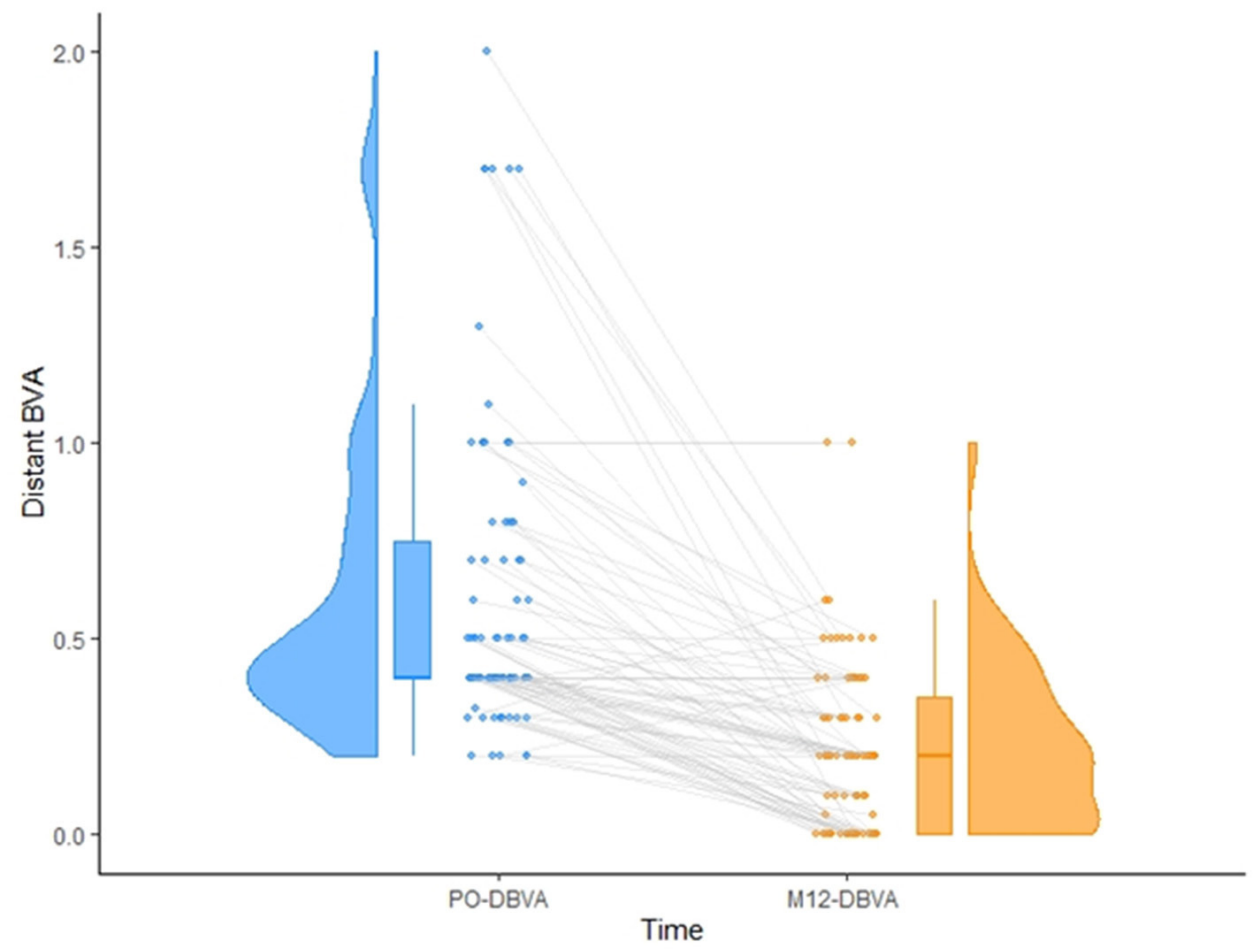
Matching between preoperative **(left)** and one-year postoperative **(right)** distant BCVA (logMAR). Representation of the inter-individual variability in recovery is also given.

Of the patients considered, 103 eyes met the inclusion criteria. Of these patients, 7 were excluded from the analysis because of ocular comorbidities during the 1-year follow-up period (2 patients with increasing cataract who had cataract surgery during the follow-up period, 4 patients with long-term ocular hypertension requiring additional medication, one with herpes simplex virus keratitis), and 13 due to missing data. Therefore, a total of 83 eyes were analyzed. The patients' preoperative features are detailed in Table 1. Analysis of correlations between the preoperative data revealed significant relationships between preoperative distant BCVA and mean CCT (*p* < 0.0001), and between preoperative distant BCVA and mean CMT (*p* = 0.01). No other significant correlations were found. One year after surgery, the mean distant BCVA was 0.2 ± 0.18 logMAR, with 63 patients (75.9%) presenting a visual recovery better than 0.4 logMAR (Figure 1). Other postoperative data are detailed and compared with preoperative values in Supplementary Table 1.

**Table 1:** Preoperative patients' characteristic and the procedure.

**Age (y)**	**72.08± 9.00**
**Sex ratio (m/f)**	29/54
**Preoperative data**	
Distant BCVA (LogMAR)	0.61 ± 0.40 [0.2–2]
Near BCVA (LogMAR)	0.63 ± 0.32 [0.1–1.2]
Spherical equivalent (D)	0.23 ± 1.56 [−7.75–6.25]
Intraocular pressure (mmHg)	13.76 ± 3.03 [8–20]
Central corneal pachymetry (μm)	651.91 ± 60.84 [520–800]
Mean foveal thickness (μm)	297.84 ± 64.89 [184–589]
Crystalline/pseudophakia x (%)	25 (30.12)/58 (69.88)
Diabetes x (%)	11 (13.25)
Morning fog x (%)	66 (79.52)
Photophobia x (%)	62 (74.70)
Epitheliopathy x (%)	23 (27.71)
Macular Edema x (%)	16 (19.28)
**Surgery**	
Graft's endothelial cell density (/mm^2^)	2,509.27 ± 204.46 [2,100–3,140]
Age of donor (years)	69.40 ± 11.17 [33–89]
Elapsed time after diagnosis (months)	4.25 ± 2.60 [1–14]
Single DMEK / combined DMEK+phaco x (%)	62 (74.70) / 21 (25.30)
Rebubbling x (%)	25 (30.12)

Quantitative data are presented as mean ± SD [min–max].

### Univariate analysis of correlations

**Figure 2:**
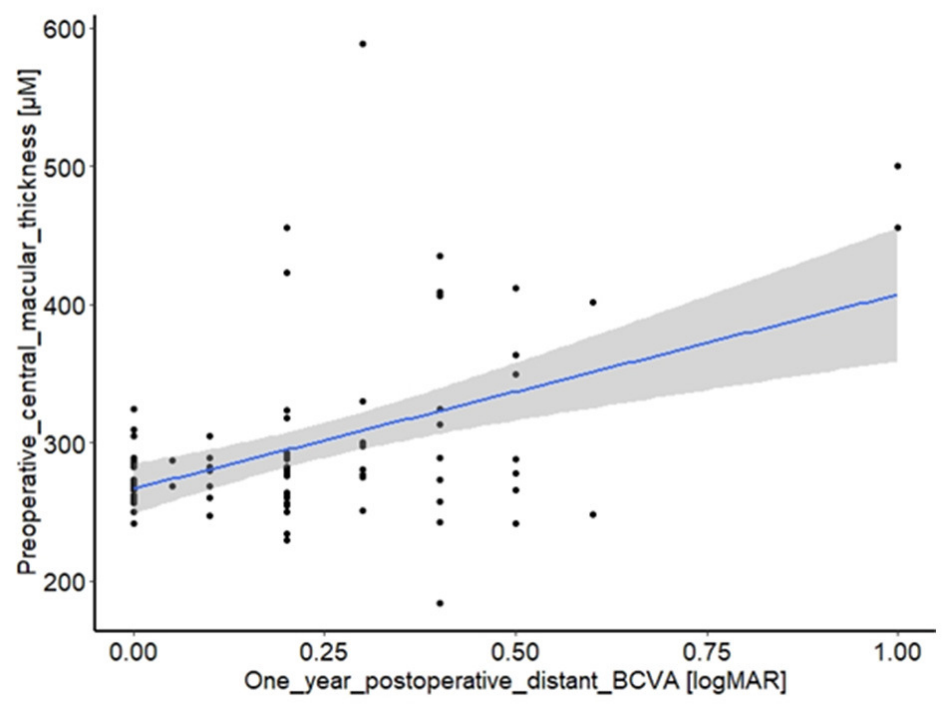
The main effect of the univariate model. Correlation between preoperative central macular thickness (μm) and one-year postoperative distant BCVA (logMAR), *p* < 0.001.

Bad visual recovery was significantly associated with an increase in preoperative macular thickness (*p* < 0.0001), and bad preoperative BCVA (*p* = 0.003). In addition, the presence of epitheliopathy was shown to have a weak association with bad recovery (*p* = 0.047). Rebubbling appeared to be associated with poor recovery; however, this finding was not significant (*p* = 0.096). None of the other data recorded were statistically significantly correlated with long-term visual outcomes. These results are summarized in Table 2. Figure 2 details the correlation between preoperative macular thickness and 1-year visual recovery. Using Youden index method, we found a cut-off value of 313 μm for CMT: it could be a pertinent preoperative indicator for poor visual recovery when exceeding this value.

**Table 2:** Univariate analysis of predictive factors for long-term visual recovery.

	**One-year BCVA**		***P***
	**Bad**	**Good**	
**Age** (years)	69.8 ± 10.85	72.80 ±8.30	0.195
Pre oprative distant BCVA (LogMAR)	0.84 ± 0.51	0.54± 0.32	0.003
Preoperative near BCVA (LogMAR)	0.81± 0.28	0.57± 0.31	0.003
Preoperative CMT (μm)	350 ± 100.34	281 ± 36.00	< 0.001
Pachymetry (μm)	659.55 ± 73.22	649 ± 56.82	0.523
Elapsed time before surgery (months)	4.35 ± 2.73	4.22 ± 2.58	0.85
Graft donnor's age (years)	72.3 ± 8.16	68.49 ± 11.88	0.186
Graft endothelial cell density (/mm^2^)	2,529 ± 247.46	2,503.01 ± 190.68	0.623
**Gender**			
F	14 (16.87)	40 (48.19)	0.595
M	6 (7.23)	23 (27.71)	
**Diabetes**			
No	16 (19.28)	56 (67.47)	0.307
Yes	4 (4.82)	7 (8.43)	
**Morning fog**			
No	3 (3.61)	14 (16.87)	0.486
Yes	17 (20.48)	49 (59.04)	
**Photophobia**			
No	3 (3.61)	18 (21.69)	0.224
Yes	17 (20.48)	45 (54.22)	
**Epithelial damage**			
No	11 (13.25)	49 (59.04)	0.047
Yes	9 (10.84)	14 (16.87)	
**Lens**			
Pseudophakic	16 (19.28)	42 (50.60)	0.257
Phakic	4 (4.82)	21 (25.30)	
**Procedure**			
Combined (DMEK+phaco)	4 (4.82)	17 (20.48)	0.531
DMEK	16 (19.28)	46 (55.42)	
**Rebubbling**			
No	11 (13.25)	47 (56.63)	0.096
Yes	9 (10.84)	16 (19.28)	

Distant best corrected visual acuity (BCVA) is considered as good if better than 0.4 LogMAR. Data are presented as mean ± SD or *N* (%).

### Logistic regression for visual recovery

**Figure 3:**
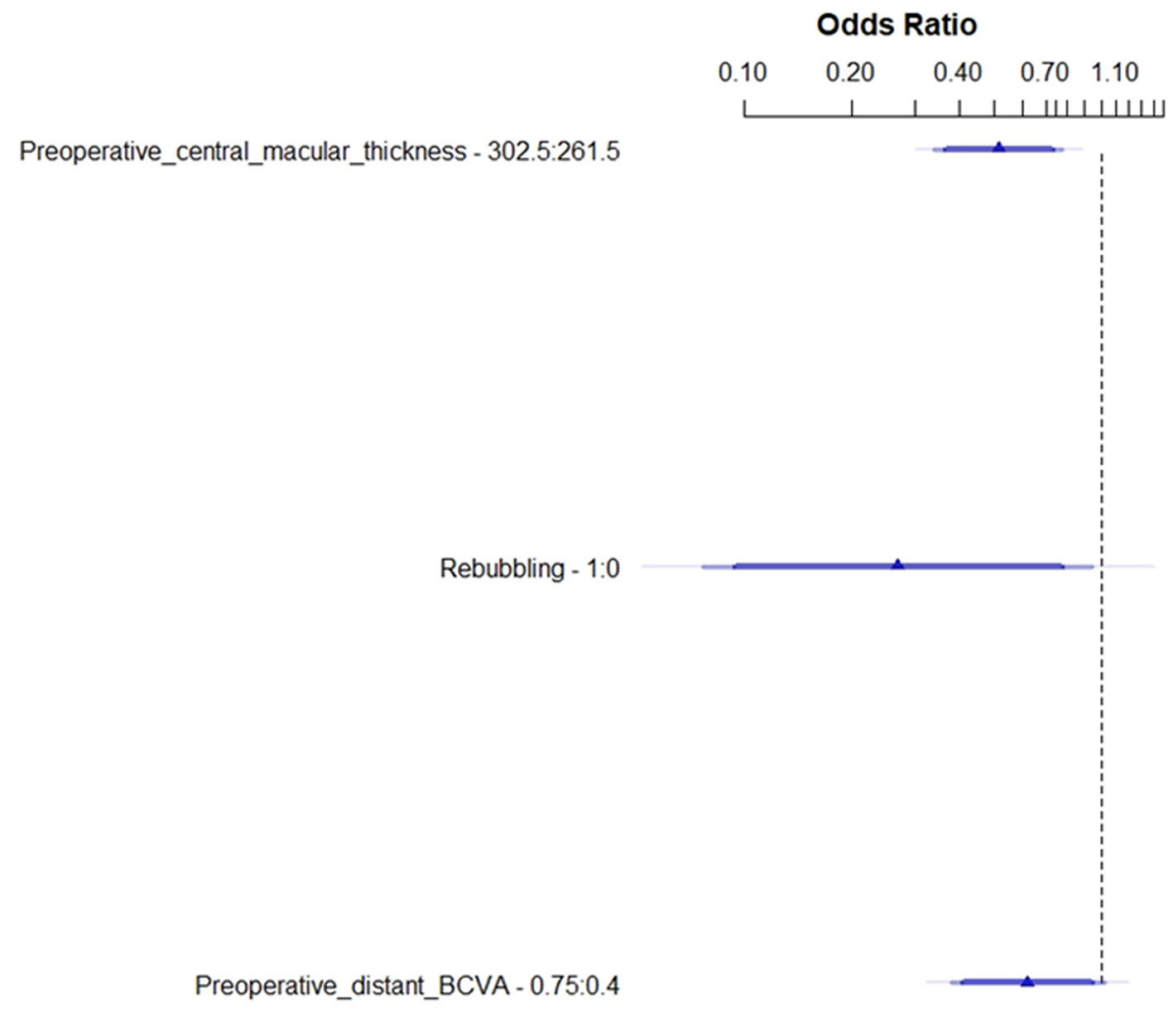
Forest plot of the main effects in the logistic regression model. Preoperative central macular thickness (OR = 0.517; IC 95% [0.342–0.781]; *p* < 0.002); rebubbling (OR = 0.269; IC 95 % [0.076–0.949]; *p* = 0.041); and preoperative distant BCVA (OR = 0.622; IC 95 % [0.379–1.023]; *p* = 0.061).

**Figure 4:**
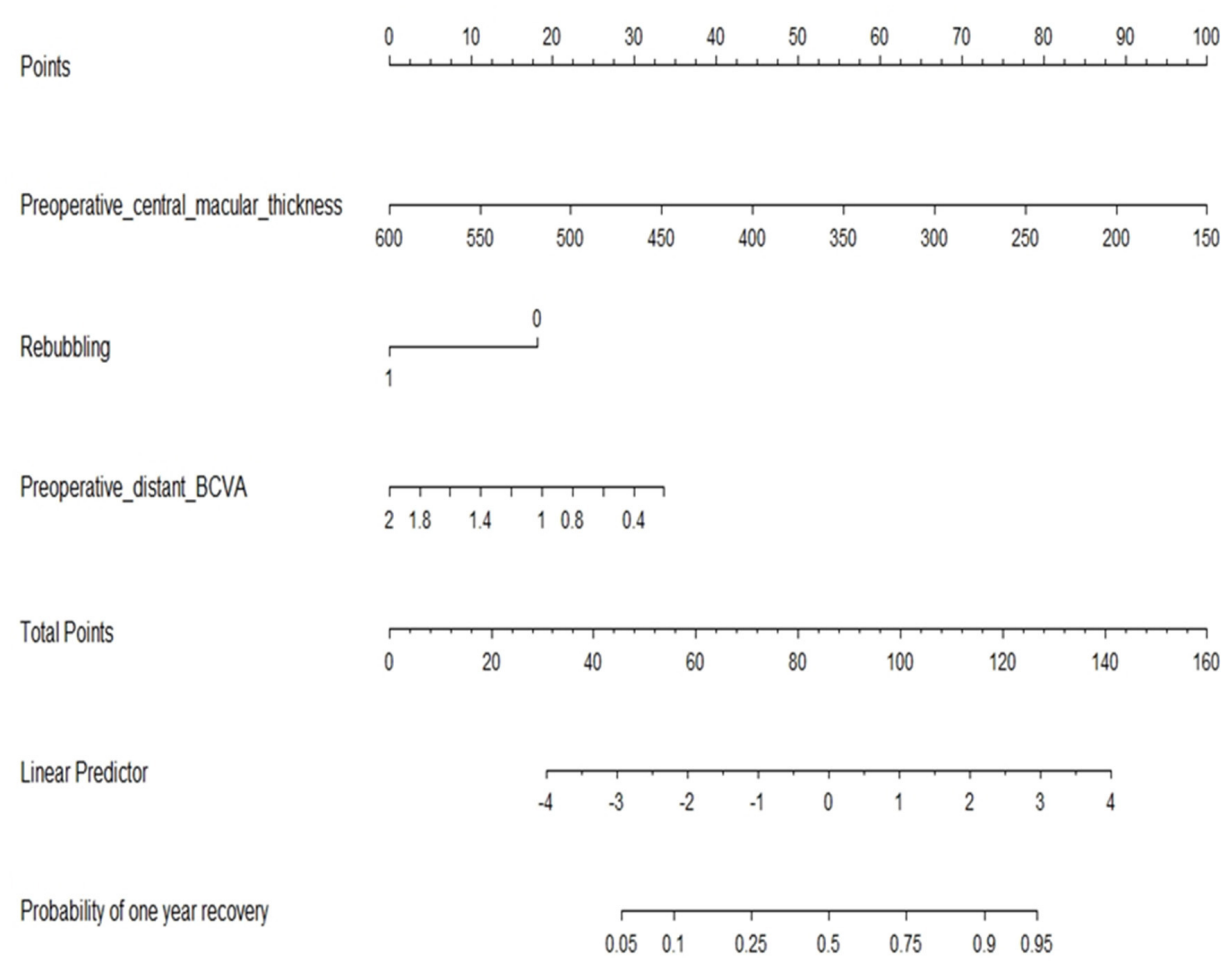
Nomogram to predict good visual recovery at 1 year. Based on logistic regression model, the nomogram includes preoperative central macular thickness (μM), preoperative distant BCVA (logMAR), and rebubbling as the main factors.

Multivariate analysis using the logistic regression model selected three main variables: the preoperative central macular thickness (*p* = 0.002), rebubbling (*p* = 0.041), and the preoperative distant BCVA (*p* = 0.062), as shown in Figure 3 and Supplementary Figure 1. Figure 3 details the odds ratio for these three preoperative parameters, which expresses the effect of each one on the probability of good visual recovery at 1 year. Our model to predict the final outcome was converted into a useable nomogram (Figure 4). Practically, following this nomogram, each variable can be converted into a subscore, and the sum of these three subscores matches the probability to recover a good BCVA after the surgery.

### Multiple linear regression to predict final BCVA

As shown in Table 3 and Supplementary Figure 2, in our final model, four factors were found to be associated with the one-year-post-DMEK BCVA, namely preoperative central macular thickness (*p* < 0.001), preoperative distant BCVA (*p* = 0.009), diabetes (*p* = 0.046), and rebubbling (*p* = 0.05). Gender also influenced slightly the visual recovery (*p* = 0.103). A formula to predict the 1-year distant BCVA has been suggested in Table 3 and can be written in an Excel spreadsheet.

**Table 3:** Multiple linear regression model for distant BCVA at 1 year.

	**Coefficient**	**IC 95%**	***P***
Gender	0.068	[−0.014–0.150]	0.103
Preoperative central macular thickness	0.001	[0.028–0.079]	< 0.001
Diabetes	0.118	[0.0024–0.233]	0.046
Rebubbling	0.084	[0.00008–0.168]	0.05
Preoperative distant BCVA	0.137	[0.012–0.083]	0.009

MODEL: BCVA [logMAR] = −0,3392621 + 0,06845741^*^ Gender (1 for female, 0 for male) + 0,001309952^*^ Preoperative central macular thinkness [μM] + 0,1179551 ^*^ Diabetes (1 for Diabetes, else 0) +0,08425589^*^rebubbling (1 for rebubbling, else 0) + 0,1372456^*^Preoperative distant BCVA [logMAR].

## Discussion

### Preoperative macular thickness as the main predictive factor for long-term visual outcome

We observed a high variability in the central macular thickness before DMEK, which was strongly linked with the visual recovery 1 year after surgery. This finding immediately raises the question of a pre-existing endothelio–macular relationship that still needs to be established. We could hypothesize that adjacent inflammation from the anterior chamber to the retina plays a role in this phenomenon, as it does in contiguous macular edema in acute anterior uveitis. This would imply anterior segment inflammation or stress during FECD. Oxidative stress has been reported to be involved in the pathogenesis of FECD. In Fuchs' dystrophy, as a consequence of a genetic add-on disorder, there is mitochondrial dysfunction leading to the potential alteration of the membrane, which, in turn, leads to excessive mitophagia (14, 15). As a result of these stress factors, structural changes appear involving fibroblastic transformation of the stromal cells and sub-epithelial fibroblast infiltration, which can be observed as a scar in other tissue.

It has already been proven that the levels of some proinflammatory cytokines, such as interleukin (IL)-1a, IL-6 IL-8, IL-17a, TNF-alpha, and IFN-c, were higher in the aqueous humor of eyes with bullous keratopathy and a low density of ECs (16, 17). In addition, high levels of cytokines seem to accelerate the loss of ECs, leading to a vicious cycle (18). As a mechanism for the development of macular edema, in this case, we could speculate that there is an interrelation between the anterior and posterior segments *via* inflammatory mediators traveling to the vitreous pocket, as could also be the case for macular edema in anterior uveitis. Therefore, in response to the diffusion of proinflammatory mediators, immunity cells could be activated, breaking the hemato-retinal barrier and leading to macular edema (19).

In the literature, some additional studies were undertaken to investigate the influence of macular edema on the outcomes of DSAEK and DMEK. However, these studies only considered the postoperative macular status. Kocaba et al. (20) studied the incidence of and risk factors for macular cystoid edema after DMEK on 80 eyes and found that postoperative macular edema was more frequent after DMEK surgery (8%) or the combined procedure, triple DMEK (18%), than phacoemulsification alone (0.1–2.3%), suggesting that DMEK is a self-risk factor. This was consistent with the results found by Heinzelman et al. (21) and Flanary et al. (22), in 155 eyes and 173 eyes, respectively. They reported macular edema after triple procedure as 13.3 and 8% and DMEK alone as 12.5 and 7.1%, respectively.

This is the first time that a study has been carried out to look for a relationship between the preoperative retinal status (central macular thickness), which may be impacted during the natural history of FECD, and subsequent visual outcomes after corneal surgery. This result is of interest in clinical practice because it could modify the indications for DMEK, suggesting that better long-term visual outcomes are achieved when the procedure is performed earlier. In addition, it could enable us to predict the visual prognosis of the eye at the preoperative visit and improve the information given to patients relating to their expected visual outcome. Once again, our results highlight the need for a close collaboration between cornea and retina subspecialists in this disease to improve the therapeutic management. Future studies could look at answering the question of whether it is possible to avoid macular thickening by adjusting the therapeutic arsenal around surgery. For instance, evaluation of the use of corticoids drops or intravitreal injection (before or during the surgery, or more than tid after) or additional non-steroidal anti-inflammatory drops would be of huge interest. Hence, the use of prophylactic anti-inflammatory medication or other drugs against macular edema in patients scheduled for DMEK with abnormal preoperative CMT should be evaluated. Moreover, we herein found a cut-off value of 313 μm for CMT related to the good/bad visual recovery, further questioning the indication of the surgery as well as the need for preoperative medication against macular edema in patients with CMT exceeding this threshold.

### Preoperative visual acuity

Statistical analysis showed that good preoperative visual acuity is strongly correlated with good recovery. This result is supported by the literature (23) and can be explained by many facts. As mentioned previously, in the advanced stages of FECD, and secondary to stress, there is fibroblastic transformation of the stromal cells and the possibility of subepithelial fibroblast infiltration. In addition, endothelial dystrophy produces a stromal haze, which creates an obvious impediment to visual rehabilitation, even after a transplant. Preoperative visual acuity is also related to ophthalmologic comorbidities, in particular, macular comorbidities, including an incipient preexistent endothelio–macular decompensation. All these factors are positively correlated with poor visual outcomes. It has to be noticed, however, that statistical analyses in the present study demonstrated that preoperative visual acuity influences long-term visual outcome in an independent way, i.e., regardless of the preoperative corneal pachymetry or macular status. Preoperative corneal pachymetry was not found to influence the visual recovery significantly.

### Rebubbling

The incidence of rebubbling in our study was 30.12%. This result is supported by the literature, 10–40% (24), 23.1% (25), and 23.8% (26). In the present study, multivariate analysis showed a significant correlation between rebubbling and visual outcomes. This was a surprising result, as it was not supported by the results reported by Gerber-Hollbach et al. (27) who studied the clinical outcomes of rebubbling 6 months after DMEK. In their study, the patients were divided into two groups: a group in which rebubbling was performed (*n* = 25) and a control group (*n* = 25). The data suggested a better global postoperative BCVA in the control group, but this difference was not statistically significant. In another study, Siebelmann et al. (24) reported that rebubbling did not influence the BCVA outcome. However, the study did not compare rebubbled eyes with a control group. Lazaridis et al. (28) showed that patients who had rebubbling in one eye and subsequently went on to have DMEK on the other eye also received a rebubbling on the second eye, with an incidence of 58.8%. The reason for this phenomenon remained unclear, but it suggests that some unidentified intrinsic factors may increase the risk of rebubbling.

In parallel, there is no consensus in the literature regarding the hypothesis of endothelial cell loss due to rebubbling. The results of Lazirids et al. (28) indicate a significant loss, while those of Feng et al. (29) do not. The literature also does not agree on whether rebubbling is a risk factor for post-DMEK macular edema. According to Inoda et al., it is recognized as a risk factor (30); however, the results of other previous studies do not support it as a risk factor for postoperative macular edema (8, 20).

We conducted an intermediate analysis—univariate and multivariate—on the 25 rebubbled eyes of our study, in order to highlight a potential associated preoperative risk factor that could suggest a bias for this result. No variable emerged from this analysis. A conclusion cannot be drawn based on this analysis, as it was obviously lacking power, and the investigation was not the main aim of our research. However, we were able to reach an agreement in recognizing that rebubbling may trigger inflammation and cause eye stress.

### Diabetes

Our results show a mild correlation in our multiple linear regression models between the incidence of diabetes and the one-year postoperative BCVA. This is supported by other studies, which showed that hyperglycemia increases the production of mitochondrial superoxide, a reactive oxygen species, leading to DNA cell damage, depleting the cellular store of antioxidants, accelerating the aging process in the cell process, and accelerating apoptosis, especially in FECD when defense mechanisms are already impaired. In addition, Zhang et al. (31) found that corneal thickness is greater in the diabetic population when compared with an aged-matched population, which was supported by other results in the literature suggesting a mild pump dysfunction. Similarly, Price et al. (32) reported that patients with diabetes experienced more endothelial cell loss after the graft than non-diabetic patients but with no visual consequences. Price also reported that diabetes status is correlated with an increased risk of rebubbling, as did Janson et al. (33). The latter studied the relationship between DMEK outcomes and diabetes in 41 patients with diabetes not on insulin therapy, 22 patients with diabetes on insulin therapy, and 271 controls. They did not show any statistically significant difference in visual acuity between the three groups at any of the postoperative time points. Last, diabetes was not shown to be a risk factor for post-DMEK macular edema in the previously quoted studies (19, 20, 29), although diabetes is still recognized as a risk factor for Irvine–Gass syndrome in cataract surgery. The reasons for these differences have not yet been clearly explained and merit further studies.

### Other factors studied

We could have speculated that procedures that included cataract surgery or other surgeries would have increased the risk of postoperative inflammation and poor visual outcomes because the procedures are longer and require more manipulation. However, our study did not show any difference in visual recovery between single or combined procedures. This result is supported by the literature, which suggests that, in terms of BCVA, DMEK and triple DMEK are comparable (34). Chaurasia et al. (35) conducted a comparative study between two groups (triple DMEK and single DMEK). They reported that triple DMEK was not associated with a higher risk of complications, i.e., there was no statistically significant difference in the incidence of graft failure, rejection, rebubbling, endothelial cell loss, or post-surgical macular edema, and BCVA improved significantly in both groups.

More interestingly, preoperative pachymetry did not show any significant association between corneal thickness and visual acuity in our study. This result has however to be balanced with the fact that individual pachymetry before Fuch's decompensation was not known. Analyzing the relationship between corneal thickening due to Fuchs' decompensation, i.e., the part of pachymetry due to corneal edema, and the visual recovery could have provided interesting information. Regarding epitheliopathy, the current study showed a marginal univariate association, which was not highly significant. There is no agreement in the literature regarding corneal thickness as a predictive factor for visual rehabilitation. Schrittenlocher et al. (7) found no correlation between corneal thickness and BCVA. In contrast, Brockmann et al. (6) studied corneal thickness as primary endpoint, and found a correlation (*p* = 0.014) between preoperative corneal thickness and visual recovery when corneal thickness was >625 μm. This result does not support the results of the current research. However, the standard deviation of the corneal thickness in the population of Brockmann's study was greater than in the current study, and we could speculate that extreme values influenced the results.

The cell density of the graft did not have a statistically significant impact on visual performance after DMEK. This result could be explained, at least in part, by the fact that grafts are carefully selected to provide enough cells, i.e., the graft's endothelial cell density is always >2,500 cells/mm^2^. Also, the other features of graft such as donor's gender and age were not statistically linked with the outcomes. Heinzelmann et al. (36), however, suggested endothelial grafts for older donors may be easier to deploy during the procedure, leading to less surgically-induced cell loss.

Last, according to the study design and exclusion criteria, the influence of other ocular factors, e.g., ocular hypertension, glaucoma, optic nerve or retinal diseases, has not been investigated.

### Limitations of the present study

First, the study could be limited by a lack of power because of the sample size. To mitigate this limitation, we used the bootstrapping method, which allowed us to validate our main result. It should also be noted that a secondary validation of our formula using a posteriori independent patients' sample would have reinforce the validation of our predictive model. Second, when collecting data, we should have taken the differential pachymetry—i.e. the proportion of corneal thickening caused by FECD—into account. However, pre-disease pachymetry measurements are difficult to obtain as patients often only consult in the advanced stages of the disease. Therefore, pre-disease pachymetry results are unknown in this study. Another study that collects differential pachymetry measurements is required to confirm the results of the present study. Finally, we did not measure cell endothelial density before and after the surgery, which could have brought some additional data. However, the difficulty to measure accurately endothelial cell density in eyes with corneal edema often leads to unproductive/biased data.

There is still variability in the long-term visual recovery after DMEK, despite our expertise and the overall excellent outcomes of this procedure. Our study highlights, for the first time, the crucial role of the preoperative macular status, which may be intrinsically linked to the natural history of FECD. Herein, the central macular thickness has been shown to be the most significant predictive factor for long-term visual outcomes, and may explain the poor recovery that is observed in some cases. Finally, this study enabled us to provide an equation to predict visual recovery after DMEK by considering some specific factors. This is of interest to practitioners as it allows them to provide appropriate and individualized information for patients concerning prognosis. Also, it questions the need for adjusting perioperative therapy according to the initial macular status to optimize the outcomes.

## Data availability statement

The original contributions presented in the study are included in the article/Supplementary material, further inquiries can be directed to the corresponding author.

## Ethics statement

The studies involving human participants were reviewed and approved by Ethics Committee of the University Hospital of Reims, Reims, France. The patients/participants provided their written informed consent to participate in this study.

## Author contributions

Design of the study: AD, ZD, and PF. Conduct of the study: CM, ED, PF, and AD. Collection and management of the data: CM, ED, and AD. Analysis and interpretation of the data and review and approval of the manuscript: AD, ZD, and CA. Preparation of the manuscript: CM and AD. All authors contributed to the article and approved the submitted version.

## Supplementary material

Supplementary Figure 1 The main effects of the logistic regression model, including pre-operative central macular thickness (5m) (p = 0.002), pre-operative distant BCVA (logMAR) (p = 0.062), and rebubbling (p = 0.041).

Supplementary Figure 2 The main effects of the multiple linear regression, including preoperative central macular thickness (5m) (p < 0.001), preoperative distant BCVA (logMAR) (p = 0.009), rebubbling (p = 0.05), diabetes (p = 0.051), and gender (p = 0.103).
