# Supplementary figures and images for: Identifying predictive factors for long-term visual recovery after corneal endothelial keratoplasty in Fuchs' dystrophy: Potential interaction between the corneal dysfunction and retinal status
Source: Front Med (Lausanne). 2023 Mar 9;10:1120283. doi: 10.3389/fmed.2023.1120283 (PMC10034073; doi:10.3389/fmed.2023.1120283)

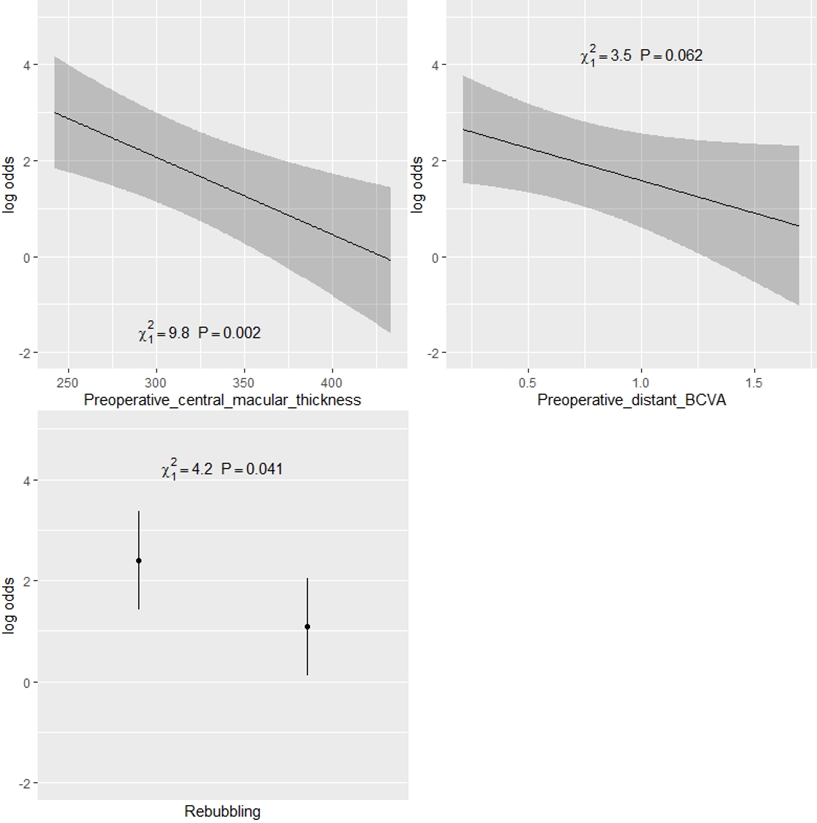

Supplement: Supplementary Figure 1 — The main effects of the logistic regression model, including pre-operative central macular thickness (5m) (p = 0.002), pre-operative distant BCVA (logMAR) (p = 0.062), and rebubbling (p = 0.041). [file Image_1.TIF]

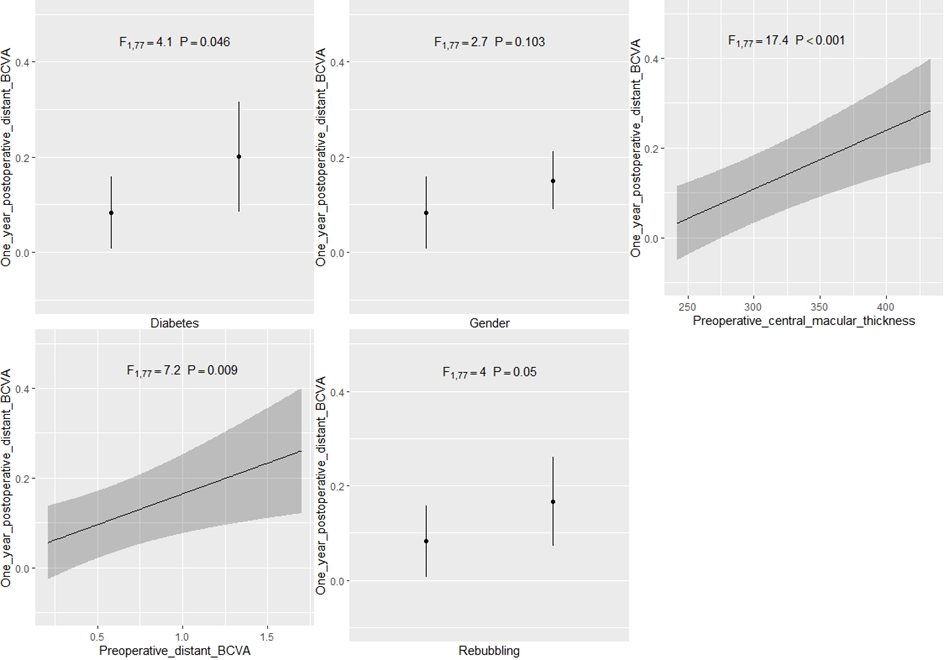

Supplement: Supplementary Figure 2 — The main effects of the multiple linear regression, including preoperative central macular thickness (5m) (p < 0.001), preoperative distant BCVA (logMAR) (p = 0.009), rebubbling (p = 0.05), diabetes (p = 0.051), and gender (p = 0.103). [file Image_2.TIF]
