# Supplementary material for: Identifying predictive factors for long-term visual recovery after corneal endothelial keratoplasty in Fuchs' dystrophy: Potential interaction between the corneal dysfunction and retinal status
Source: Front Med (Lausanne). 2023 Mar 9;10:1120283. doi: 10.3389/fmed.2023.1120283 (PMC10034073; doi:10.3389/fmed.2023.1120283)
Supplement: Supplementary file 3 [file Table_1.docx]

***Supplementary Table 1:*** One-year postoperative data. Quantitative data are presented as mean ± SD [min – max]. Comparisons with preoperative values are performed using paired T-test or Chi-square test.

|  |  | p |
| --- | --- | --- |
| Distant BCVA (LogMAR)  Near BCVA (LogMAR)  Spherical equivalent (D)  Intraocular pressure (mmHg) | 0.22 ± 0.16 [0 – 1]  0.23 ± 0.20 [0 – 1]  0.30 ± 1.11 [-7 – 4.125]  14.03 ± 3.25 [6 – 21] | <0.0001  <0.0001  0.02  0.64 |
|  |  |  |
| Central corneal pachymetry (µm) | 516.73 ± 32.12 [415 – 600] | <0.0001 |
| Mean foveal thickness (µm) | 308.38 ± 51.44 [195 – 807] | 0.39 |
| Macular edema x (%) | 19 (22.89) | 0.57 |
